# Local setting influences the quantity of household food waste in mid-sized South African towns

**DOI:** 10.1371/journal.pone.0189407

**Published:** 2017-12-12

**Authors:** Gamuchirai Chakona, Charlie M. Shackleton

**Affiliations:** Department of Environmental Science, Rhodes University, Grahamstown, South Africa; University of Notre Dame, UNITED STATES

## Abstract

The world faces a food security challenge with approximately 868 million people undernourished and about two billion people suffering from the negative health consequences of micronutrient deficiencies. Yet, it is believed that at least 33% of food produced for human consumption is lost or wasted along the food chain. As food waste has a negative effect on food security, the present study sought to quantify household food waste along the rural-urban continuum in three South African mid-sized towns situated along an agro-ecological gradient. We quantified the types of foods and drinks that households threw away in the previous 48 hours and identified the causes of household food waste in the three sites. More households wasted prepared food (27%) than unprepared food (15%) and drinks (8%). However, households threw away greater quantities of unprepared food in the 48-hour recall period (268.6±610.1 g, 90% confidence interval: 175.5 to 361.7 g) compared to prepared food (121.0±132.4 g, 90% confidence interval: 100.8 to 141.3 g) and drinks (77.0±192.5 ml, 90% confidence interval: 47.7 to 106.4 ml). The estimated per capita food waste (5–10 kg of unprepared food waste, 3–4 kg of prepared food waste and 1–3 litres of drinks waste per person per year) overlaps with that estimated for other developing countries, but lower than most developed countries. However, the estimated average amount of food waste per person per year for this study (12.35 kg) was higher relative to that estimated for developing countries (8.5 kg per person per year). Household food waste was mainly a result of consumer behavior concerning food preparation and storage. Integrated approaches are required to address this developmental issue affecting South African societies, which include promoting sound food management to decrease household food waste. Also, increased awareness and educational campaigns for household food waste reduction interventions are discussed.

## Introduction

The world faces a food security challenge with approximately 868 million people undernourished and about two billion people suffering from the negative health consequences of micronutrient deficiencies [1]. Yet, at least one-third of food produced for human consumption is lost or wasted along the food chain between farm and fork [2–7]. Food waste refers to wholesome edible material intended for human consumption, arising at any point in the food supply chain that is instead discarded, lost, degraded or consumed by pests [8]. Food loss is defined as the decrease in food quantity or quality which makes it unfit for human consumption [9]. According to the European Commission [10], food waste is composed of raw or cooked food materials such as vegetable peelings, meat trimmings and spoiled or excess ingredients or prepared food as well as bones, carcasses and organs. However, food waste can be measured only for edible products that are directed to human consumption [6]. Food losses take place at production, postharvest and processing stages in the food supply chain and the food losses that occur at the end of the food chain (retail and final consumption) are called “food waste” [11]. In our study food waste refers to food losses that occur at the end of the food chain (final consumption at household or consumer level) which include edible products that are directed to human consumption and are discarded when not consumed for various reasons. It is related to consumers’ behaviour [6,11] and it includes food loss before, during or after meal preparation in the household.

According to Gustavsson et al. [6], the annual value of wasted food along the whole food chain is approximately US$ 680 billion in industrialised and US$ 310 billion in developing countries. About 31–40% of all the food produced in the United States is never eaten [12,13], and in the United Kingdom, consumers discard about one-third of the food they purchase even though more than 60% is still suitable for human consumption [14]. Despite considerable efforts to help in reducing household food waste over the last three years in the United Kingdom, household food waste has increased to 7.3 million tonnes in 2015 compared to 7.0 million tonnes in 2012 [15]. The amount of avoidable household food waste (i.e. the food that could have been eaten) also increased by 5.1% in 2015 [15]. It has been estimated that globally the amount of food wasted is four times the amount needed to eliminate world hunger [6]. Thus, strategies to avoid and reduce food waste could go a long way towards achieving world food security [3,6]. Reducing food waste would also contribute positively to biodiversity and ecosystem services conservation through reduced land transformation and use of chemicals, reduced greenhouse gas emissions, and the costs of waste disposal and processing could be saved and redirected [6,10].

The types and quantities of food wasted vary between and within countries, as well as between households. At the macro-scale, the type and quantity of food waste differs between developed and developing countries. In developed countries, most (approx. 60%) food waste is generated after it has been purchased by consumers [6,10,16]. In contrast, in developing countries most food is lost before it reaches the final consumer, i.e. in the growth, storage and distribution phases [6], resulting from financial, managerial and technical constraints in harvesting techniques as well as insufficient storage and cooling facilities. The quantities also differ, with means of 95–115 kg/year/person in North America and Western Europe, compared to 6–11 kg/year/person in sub-Saharan Africa and South/Southeast Asia [6]. Other macro-scale factors may include location, such as rural sites compared to urban ones, and global or national food prices. Typically, urban households waste more food than their rural counterparts [17] because of higher wealth and their need to store food at home (after purchase) rather than harvesting it on demand as occurs during the growing season in rural settings. This pattern may also result in differences between rural areas, based on the degree of suitability for agricultural production, and the length of the growing season. Urbanisation is a primary driver of changes in dietary transition, consumption patterns, and hence also food waste patterns [11]. Lundqvist et al. [3] reported that dietary transition typically leads to increased consumption of food that has a short shelf-life, such as dairy, fruit and vegetables, which may result in greater food waste in the absence of efficient storage options in the home.

At the household scale, the quantities of food waste depend on a range of factors such as household size, composition, income, demographics and culture [11]. For example, work in developed countries indicates that larger households waste less food per capita than smaller households [8,18–19]. This relationship varies between households with children and those without, as generally, young people waste more food than older people [20–21]. Low income households are presumed to waste less food than wealthier households [21], although Parfitt et al. [11] note that some studies report little or no correlation between income and food wastage. At times, this may also be a covariate with culture or ethnicity. For example, Hispanic households in the USA waste approximately 25% less food than non-Hispanics, but this may also be related to wealth [11], whilst Rathje and Murphy [22] suggest it is also a reflection of cooking styles as Hispanic cuisine involves a lot of mixed dishes to which it is easy to add leftovers. These household level influences on food waste have rarely been properly disaggregated in developing countries, including South Africa.

Indeed, there is very little knowledge or empirical quantification of household food waste in sub-Saharan African countries, including South Africa [23–24]. This lack of empirical information led Nahman et al. [25] to estimate household waste patterns from secondary data of waste stream analyses in landfills of large South African cities characterising the relative contribution of food waste to the overall waste stream. However, this approach has several inaccuracies. On one hand, food waste production was underestimated as not all wasted food goes to landfills, but rather may be dumped on compost heaps or fed to livestock or domestic animals. On the other hand, the organic component on landfills also includes garden waste (accumulated plant matter from gardening activities such as cutting the lawn, weed removal, hedge trimming or pruning consisting of lawn clippings, leaf matter, wood and soil), which is not always easy to separate from food waste [26]. Using similar secondary data sets Nahman and De Lange [26] considered the entire food chain, whilst Oelofse and Nahman [23] considered the food value chain based on assumptions from surveys in other countries [23], thus none are based on empirical measures at the household level, and none considered rural settings. Although, some studies in South Africa have shown that consumer food waste contributes significantly to the waste stream [27–28], no national data on food waste is available for the country [25]. Indeed, little is known about the quantities of food waste generated by households in South Africa, or how it varies by location (such as urban or rural) or household wealth. Food waste research is neglected yet it is an important aspect of the food system [24] as it negatively affects food security [29]. South Africa has high rates of under- and mal-nourishment [30] and hence integrated approaches are required to address this developmental issue affecting South African societies, which include promoting sound food management to decrease food waste.

As food waste has a negative effect on food security, and because there is little empirical information on household food waste patterns in sub-Saharan African countries, including South Africa, this study sought to quantify and characterise household food waste in different settings. We did so by surveying rural and urban households at three sites along a macro-scale gradient of agro-ecological potential (geographical areas exhibiting climatic conditions that determine their ability to support rain-fed agriculture). This allowed analysis of differences between sites along the agro-ecological gradient, between households along the rural-urban continuum and in relation to household attributes such as household wealth, size, food expenditure and household food insecurity access scale (HFIAS). We hypothesised that: (1) The quantities of household food waste would decrease with declining potential for agriculture because in rain-fed agricultural areas, weather conditions as well as constraints in harvesting techniques, insufficient storage and cooling facilities may promote the quantities of food waste, (2) Households in rural areas would waste less food than those in the urbanised settings because urban households have been reported to waste more food than their rural counterparts [17] because of higher wealth and their need to store food at home (after purchase) rather than harvesting it on demand as occurs during the growing season in rural settings, (3) Wealthy households would throw away more food than poor households because poor households may not have “enough” food to spare hence are compelled to cut down on the food they waste. This can also follow reports by [21] that low income households are presumed to waste less food than wealthier households, (4) Most food waste would be from unprepared or raw food which could be attributed to poor storage facilities in homes or behavioural condition of hording food when being sold at low prices, (5) Households with limited access (having difficulties in getting food) to food would waste less food than those with good access to food because having limited access to food may create an environment where consumers are compelled to prepare what is enough for the meal and may not have more to spare and discard, and (6) Single households would waste more food on a per capita basis. Smaller households would throw away more food per capita than larger households as the amount of food waste generated per person decreases with increasing households size and this has also been reported in developed countries [11,18–19, 31].

## Materials and methods

### Study sites

**Fig 1:**
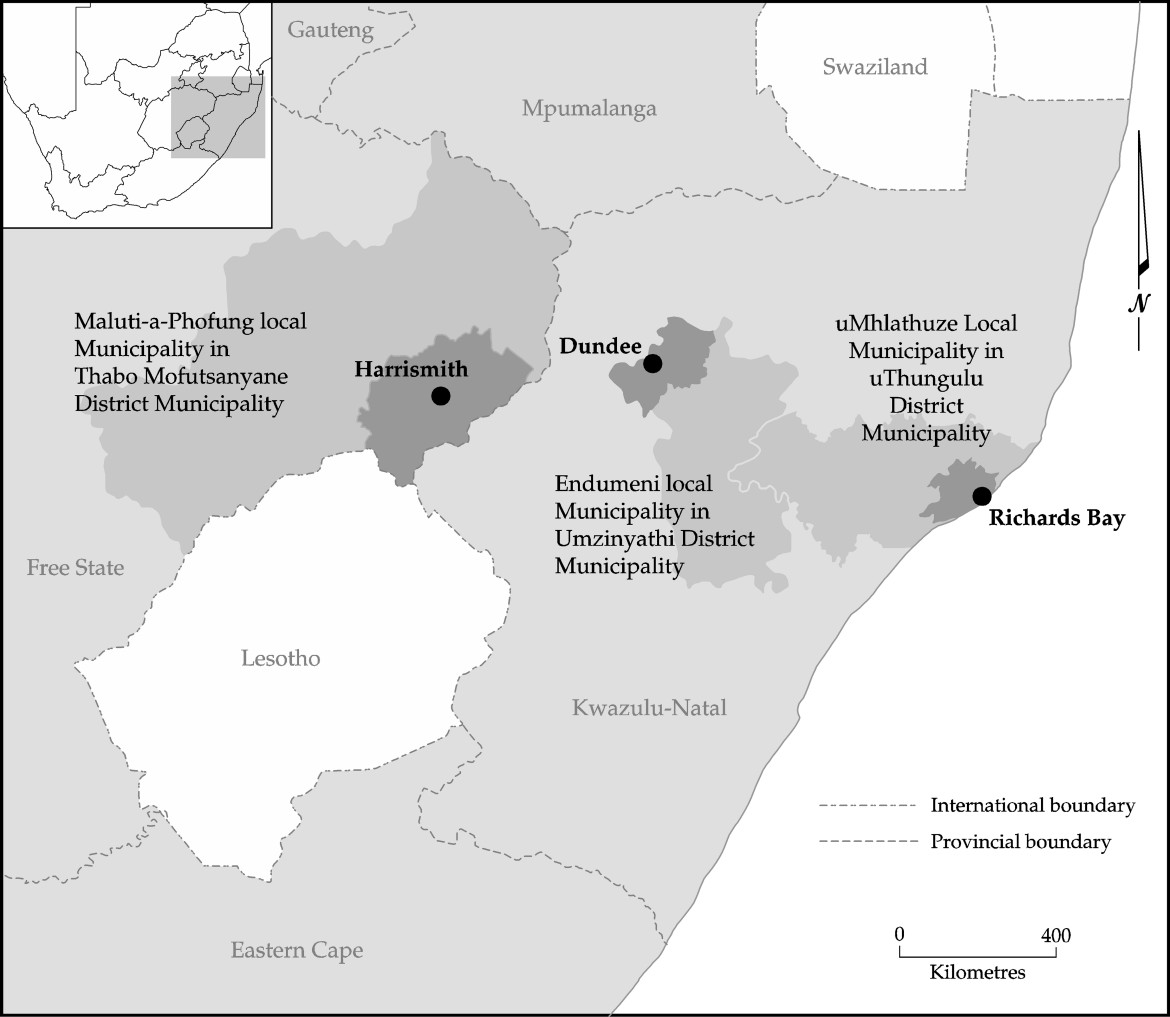
Location of study towns in South Africa.

This study was conducted in three medium-sized (35 000–50 000 people) towns in South Africa; Richards Bay and Dundee in KwaZulu-Natal province and Harrismith in the Free State province (Fig 1).

**Fig 2:**
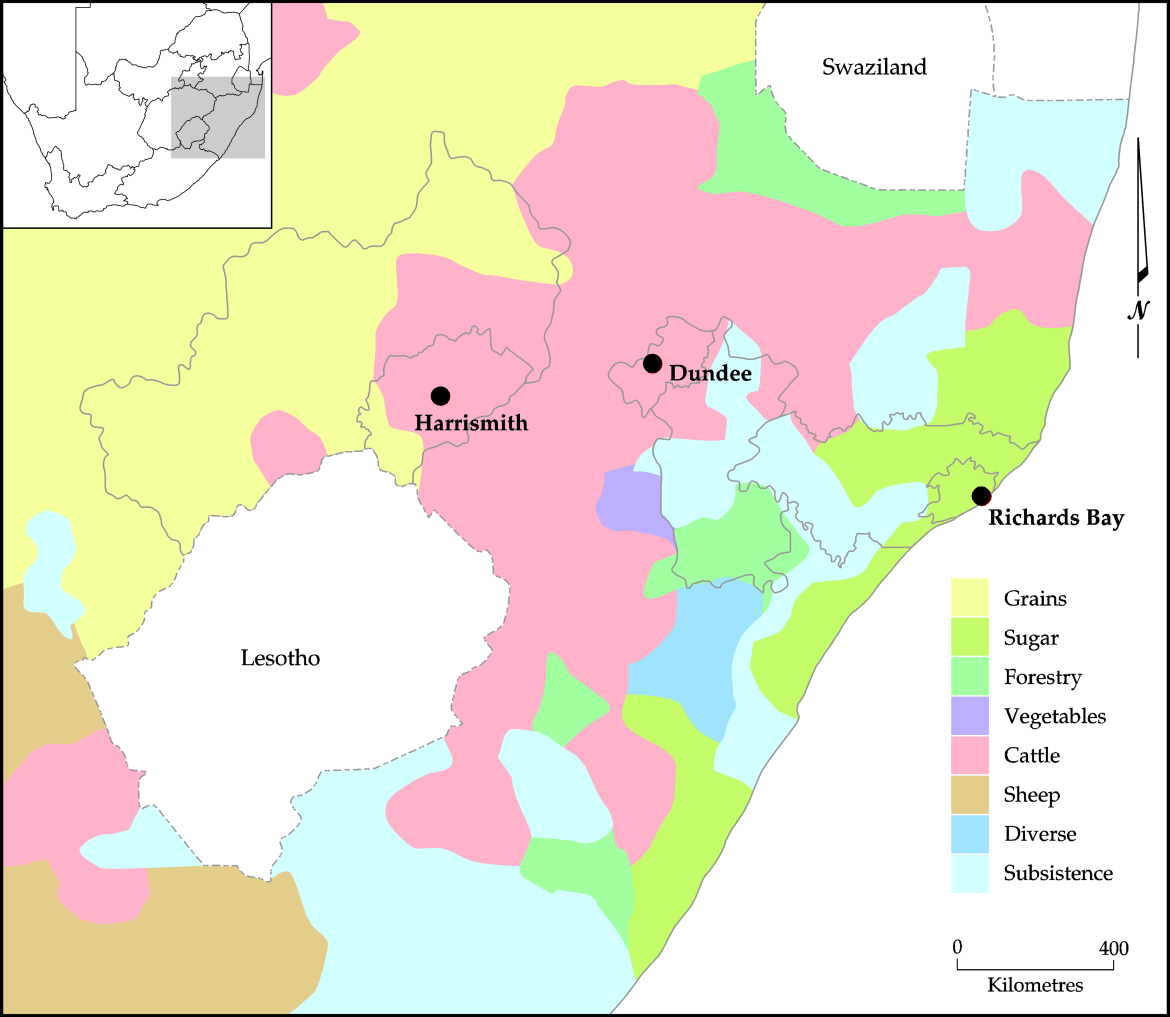
South Africa’s agricultural regions.

Agro-ecological zones (AEZs) are geographical areas exhibiting similar climatic conditions that determine their ability to support rain-fed agriculture. These are influenced by latitude, elevation, and temperature, as well as seasonality, rainfall amounts and distribution during the growing season [32]. The towns were selected along a gradient of agro-ecological suitability, with Richard’s Bay being a warm (mean annual temperature is 21.5°C) coastal and high rainfall site (approximately 970 mm per annum (p.a.)), while Harrismith is central and relatively high altitude (1 650 metres above sea level (m.a.s.l)), temperate (mean annual temperature is 18.6°C) with low rainfall (approximately 622 mm p.a.) and Dundee being intermediate (inland, 1 260 m.a.s.l; 14.2°C and 683 mm p.a.). The seasonality of the rainfall increases along this gradient, along with the severity of winter temperatures. Thus, the gradient also reflects one of declining suitability for rain-fed agriculture, from high in Richards Bay to low in Harrismith where rural farms mostly practice cattle ranging. The agricultural regions of South Africa are shown in Fig 2 where Richards Bay falls in the region that specialises mostly in sugarcane production whilst cattle ranching is mostly suitable in Dundee and Harrismith. Each site included the rural, peri-urban and urban complex and data were collected along a rural-urban continuum. Unemployment is high (>30%) in all sites, but higher in the rural zones than the urban ones [33,34].

### Sampling

Food waste data was obtained through administering questionnaires to randomly selected households at each site. Within each town, 200 households were randomly selected, comprising of 60 rural households, 80 peri-urban households and 60 urban households. Random cluster sampling using ArcGIS was used with five randomly selected households per cluster. There were twelve clusters in each of the urban and rural areas and 16 clusters in the peri-urban area. GPS coordinates for the households were generated within each selected cluster. A woman of reproductive age (15–49 years old) and the person in the household who prepares most of the meals was interviewed as she was regarded as having the knowledge of all the food that was consumed and not consumed within the household. All interviews were conducted face-to-face after the researcher had gained consent from the interviewee and it took an average of 1hr 45 minutes to finish the interview. However, not all selected households agreed to participate in the interviews, therefore those which refused were left out, leaving a total of 554 households with 183 individuals interviewed in Richards Bay; 173 in Dundee and 198 in Harrismith. The three towns were regarded as having equal weight during analysis in this study as it was difficult to get participants.

Data were collected between October and November 2014. A questionnaire was used to collect information on households’ behaviour regarding the meals they consume at home (S1 File). That is, general information on where household members usually eat their meals, if most household members usually ate the same meal, how often some food was not eaten and what households did with the left-overs. In the present study, food waste was classified into three main classes which were prepared food waste, unprepared food waste and drinks waste. Participants were asked to report on the food waste that they had generated over the past 48 hours in their homes, including classifying the type of food waste, naming the type of food, the quantity (weight or volume) and reason for disposal. Participants were also asked to measure and record their food waste as they created it during the 48-hour period before the interview using estimated standard household measures such as cups, tablespoons or teaspoons. Prepared and unprepared foods were measured in kilograms (kg) and grams (g) whilst drinks were measured in litres (l) and millilitres (ml). The study also used a conversion factor of 1 litre = 1 kg considering that 1 cubic metre is equal to 1000 litres or 1000 kg. However, it was not possible for households to separate some of the prepared foods and weigh them separately according to food type and therefore these were measured as mixed dishes.

Households were also given food waste diaries where they were asked to record the food they threw away, specifying the type of food, quantity and their reasons for throwing that food away. Upon every reason, they were asked to write their feelings when throwing away the food and their perceptions about food waste. Unfortunately, this method did not yield any data as none of the households returned the diaries upon collection. However, these measurements need to be taken with caution as it has been found that the above-mentioned methods can yield data that are not representative of habitual household behavior on wasting food. For example, Quested et al. [35] found that quantities of waste recorded in diaries are approximately 40% lower than those obtained from analysis of waste streams and Hanson et al. [36] also reported that household food waste diary approaches systematically underestimates food loss and waste levels.

Additionally, the internationally applied Household Food Insecurity Access Scale (HFIAS) was performed. The HFIAS score is a continuous measure of the degree of food insecurity mostly related to access in the household in the past four weeks (30 days). The HFIAS tool is composed of nine questions that ask about any possible modifications households made in their diet or food consumption patterns due to limited resources to acquire food [34]. The nine questions are subdivided into three themes of food insecurity which are: 1) experiencing anxiety and uncertainty about the household food supply; 2) insufficient quality of diet which includes variety and preferences of the type of food and 3) insufficient food intake or reducing quantity of food consumed [37]. The questions address the situation of all household members and do not distinguish adults from children or men from women or adolescents.

The nine questions represent a generally increasing level of severity of food insecurity and nine “frequency-of-occurrence” questions were asked as a follow-up to each occurrence question to determine how often any condition occurred. For each frequency-of-occurrence question, a score was assigned to each household: 1 if the response was rarely (condition having happened once or twice in the past four weeks); 2 if it occurred sometimes (three to ten times in the past four weeks) or 3 if the answer was often (occurred for more than ten times in the past four weeks).

Households were assigned a score that ranged from 0 to 27 at the end of the nine questions which was based on their response to the nine questions (yes or no) and frequency-of-occurrence (rarely, sometimes and often). A household was assigned a score of zero if the household responded “no” to all occurrence questions. The maximum score of 27 was given to a household if the response to all nine frequency-of-occurrence questions was “often”, and scores were added together. A high HFIAS score indicates household’s poor access to food and significant household food insecurity [37].

Information on household characteristics such as the household size, age, gender of household head, sources of food, income, land acquisition, wealth (assets acquired by household) and the cost of food purchases per week were also asked. An index of wealth was created by combining information obtained on the household’s possessions and this included car/truck, motorbike, tractor, bicycle, fridge, television, radio, cattle/goats, chickens, cell phone, house and electricity. For each household, the number of each asset was normalised (by dividing with the highest number obtained in each category for all households) then all summed to get a wealth index per household, which could range from zero to 12.

All interviews were conducted in the respondent’s preferred language of isiZulu in Richards Bay and Dundee, and Sesotho in Harrismith or English. Different enumerators were used in each town and in all study sites, they were trained on how to conduct interviews using the questionnaire so as to provide full understanding of the administered questions. Ethics approval was obtained from the Rhodes University Ethics Committee, and all the respondents signed informed consent forms after the researcher had explained the details of the project to them (S2 File).

### Statistical analysis

Data were entered and cleaned using Microsoft Excel and all statistical analyses were performed using Statistica version 13 (StatSoft Inc.). A 2-way ANOVA was conducted to measure if there were any significant differences in the amounts of food waste generated by households between and within towns and clusters as interviewers had different clusters. No Significant differences were observed and therefore data from all interviewers was used for analysis. Descriptive data is presented as means and standard deviations (SDs) (mean ± SD), standard error of mean (SEM), confidence intervals (CI) as lower confidence limit (LCL) and upper confidence limit (UCL) and percentages. Considering the random cluster sampling used in this study, all household observations for food quantities within a cluster were simply reduced to a single summary measure, which was the cluster mean and then standard statistical methods were used to analyze these cluster means as if they were the primary observations. The mean amount of food wasted (including prepared food, unprepared food and drinks) by households within 48 hrs was calculated from the amounts reported by households. This was used to calculate the amount which was wasted by households over a year and the amount wasted per household over a year was divided by the mean household size of the sample, to get the estimated amount which was wasted per person per household over a year period. The differences in amount of food wasted per household between towns and locations were tested using 2-way ANOVA and differences within each town were tested using 1-way ANOVA. Post-hoc followed in case a significant effect was detected. Post-hoc tests (the Bonferroni correction) was performed when the equality of variances’ assumption holds and this also provided specific information on which means were significantly different from each other. The associations of the food waste quantities with food expenditure, household size, wealth variables and HFIAS were examined through Spearman correlation tests and the amount of food waste was used as a response variable. Statistical significance was set at p < 0.05 for all tests. The South African Rand to US dollar exchange rate was approximately 11:1 at the time.

## Results

### Household characteristics

The full sample consisted of 554 women of reproductive age with a mean age of 31.5±10.0 years (90% CI: 30.9 to 32.3 years). The household size for the full sample was 6.9±3.89 persons (90% CI: 6.7 to 7.2 persons) and almost 60% of the households were female-headed. More than 80% of households in Dundee and Harrismith received some form of cash income whilst only 59% of household in Richards Bay did so. Households in Richards Bay were spending less cash per week on purchasing food (R196±180) than in Dundee and Harrismith with R333±253 and R323±271 per week, respectively, on food. The wealth index was almost similarly low for all towns, ranging between 2.3±1.0 in Dundee and 2.6±0.6 in Richards Bay. About 73% of households in Richards Bay had land available for their own production whilst only 57% of households in Dundee and 27% in Harrismith did so.

### Household behaviour regarding meals at home

In all three towns, the majority of households reported that household members were always eating their meals at home and were eating the same meal at the same time (Table 1). Household members in all three towns rarely had their meals apart from each other and not at home.

**Table 1:** Comparison of the eating patterns of households in the three towns.

Town	Households eating meals together^1^	Always at home^2^	Mainly at home^3^	Partly at home and partly elsewhere^4^	Mainly elsewhere^5^	Always elsewhere^6^
Richards Bay	82	78	8	9	4	1
Dundee	92	86	4	7	2	1
Harrismith	80	77	9	9	4	1
Grand mean	84	80	7	9	3	1

^1^Percentage of households eating meals together.

^2^Percentage of households that always eat at home.

^3^Percentage of households that eat mainly at home.

^4^Percentage of households that eat at home and partly elsewhere.

^5^Percentage of households that eat mainly elsewhere.

^6^Percentage of households that always eat elsewhere.

For all meals in all towns, households rarely left any food after the meal (Table 2). Only a very small percentage of the households has reported to have left food uneaten at each meal.

**Table 2:** Percentage of households not consuming all the prepared food and the frequency of occurrence per month.

Town	Very rarely	< 5 times per month	5–10 times per month	11–20 times per month	> 20 times per month
Richards Bay	74	11	6	3	6
Dundee	85	11	3	0	0
Harrismith	79	9	5	3	4
Grand mean	80	10	5	2	4

When the food was not consumed, households in all towns reported that they rarely throw it away but rather keep the leftovers and consume the food within a day or two (Table 3). Very rarely did households give the food to other people, feed animals or throw it away.

**Table 3:** Different ways in which households usually deal with the leftover food.

Town	Keep and eat in the next day or two	Give away to other people	Feed animals	Throw away
Richards Bay	87	6	3	4
Dundee	86	5	8	1
Harrismith	80	6	9	6
Grand mean	84	6	6	3

Note: All values in the table expressed as % of households.

### Types of food waste along the agro-ecological gradient

Overall, 191 households out of 554 sampled households (35% of the sample) had wasted food (discarded food) in the past 48 hours and 67 households (12%) were from Richards Bay, 55 households (10%) from Dundee and 69 households (13%) from Harrismith (data not reported in the tables). Overall all households, about 27% threw away prepared food, 15% threw away unprepared food whilst 8% of the households in all towns wasted drinks (Table 4). There were no significant differences in the percentages of households wasting food and drinks between the towns (prepared food (F_2, 538_ = 1.38, p = 0.253), unprepared (F_2, 538_ = 2.66, p = .071) and drinks (F_2, 538_ = 0.54, p = 0.58)).

**Table 4:** Percentage of households throwing away different types of food waste within each town in the previous 48 hours and the aggregate.

Town	Location	Prepared food waste		Unprepared food waste		Drinks	
Richards Bay	*Urban*	31	33	12	12	9	7
	*Peri-urban*		24		9		6
	*Rural*		38		16		13
Dundee	*Urban*	24	14	12	14	6	8
	*Peri-urban*		15		15		8
	*Rural*		10		10		3
Harrismith	*Urban*	25	39^a^	20	31^a^	8	22^a^
	*Peri-urban*		21^b^		15^b^		2^b^
	*Rural*		17^b^		14^b^		2^b^
Grand mean	*Urban*	27	34	15	20	8	13
	*Peri-urban*		23		13		5
	*Rural*		27		13		6

Note: All values in the table are expressed as % of households. Unlike superscripts show significant differences in the percentage of households wasting different food waste types between and within towns. Data was analysed using two-way ANOVA, prepared food (F_2, 538_ = 1.38, p = 0.253), unprepared (F_2, 538_ = 2.66, p = .071) and drinks (F_2, 538_ = 0.54, p = 0.58) (n = 554). No significant differences were observed. For prepared food in Harrismith town: pairwise comparisons by post hoc Bonferroni indicated: 1. prepared food (F_2, 195_ = 4.22, p = 0.02) in urban households (n = 55) thrown away more (p<0.05) than the peri-urban households (n = 85) and rural households (n = 58), although it was similar in latter. 2. Drinks (F_2, 195_ = 12.7, p = 0.000001) were wasted more in urban households (n = 55) than the peri-urban (p < 0.00001) (n = 85) and rural households (p < 0.00001) (n = 58). For unprepared food in Harrismith town: pairwise comparisons indicated: 1. Unprepared food in urban households (F_2, 195_ = 3.7, p = 0.03) was thrown away more (p<0.05) than the peri-urban and rural households.

Considering the rural-urban continuum, more urban residents threw away all classes of food waste relative to their peri-urban and rural counterparts (Table 4). However, the differences were significant only for Harrismith. Pair-wise comparisons (Bonferroni correction) for prepared food (F_2, 195_ = 4.22, p = 0.02) showed that urban households threw away prepared food significantly more than the peri-urban households (p < 0.05) and rural households (p < 0.05) and showed that drinks (F_2, 195_ = 12.7, p = 0.000001) were wasted significantly more in urban households than the peri-urban (p < 0.00001) and rural households (p < 0.00001).

With respect to unprepared food, there were no significant differences observed in the percentage of households wasting unprepared food although Harrismith had the highest percentage of households who were throwing away unprepared food whilst Richards Bay and Dundee had the lowest percentages (Table 4). However, the percentage of households throwing away unprepared food followed the rural-urban continuum with households in the urban locations throwing away more unprepared food than their peri-urban and rural counterparts, although not significantly so, other than in Harrismith (F_2, 195_ = 3.7, p = 0.03). Pair-wise comparisons (Bonferroni correction) for unprepared food in Harrismith showed that urban households threw away unprepared food significantly more than the peri-urban households (p < 0.05) although this was not the case for rural households.

The types of food waste were similar across the three sites. In general, the prepared foods that were most commonly wasted were pap (corn flour porridge), meat, vegetables and rice. Pap and meat were thrown away by greater than 20% of the households in all the towns (Table 5). In Richards Bay, pap and meat were mostly discarded in the rural areas, in Dundee these were mostly thrown away in the peri-urban and rural areas whilst in Harrismith it was the urban households who threw away these food items. Rice was frequently wasted by households in Richards Bay and it was mostly in the rural location, whilst vegetables were mostly wasted by households in the peri-urban locations of Dundee and Harrismith. Potatoes were mostly wasted in Harrismith whilst samp (coarsely crushed corn) was mostly wasted in Richards Bay and Dundee and beans mostly in Dundee. Bread and fish were among the least wasted prepared foods in all the towns.

**Table 5:** Percentage of households wasting different types of prepared foods per town in the previous 48 hours.

Town	N	Pap	Rice	Meat	Beans	Vegetables	Samp
Richards Bay	67	27	30	21	1	6	10
Dundee	55	29	7	22	11	22	7
Harrismith	69	25	12	22	4	26	0
Grand mean	191	27	16	21	6	18	6

The unprepared foods that were wasted by households in all towns were potatoes, fish and to larger extent vegetables (mostly tomatoes and cabbage) which were discarded by greater than 14% of the households in all towns (Table 6). Vegetables were mostly wasted in the rural location in Richards Bay (6%), whilst in Dundee (13%) and Harrismith (13%) it was the peri-urban dwellers who wasted more vegetables. Fish and meat were mostly wasted in Harrismith urban with 10% of households throwing away these unprepared foods. The other unprepared foods that were wasted were town specific. For example, samp, maize meal and flour were wasted in Richards Bay only (mostly peri- urban and urban locations), whilst fruits were thrown away in Dundee only (more in the urban location). Unprepared waste from beans, bread and meat were recorded in Harrismith and Dundee only whilst that from rice was found in Richards Bay and Dundee only.

**Table 6:** Percentage of households wasting different types of unprepared foods per town in the previous 48 hours.

Town	N	Meat	Vegetables	Potatoes	Beans	Fish	Bread	Fruits	Rice
Richards Bay	67	0	15	4	0	4	0	0	3
Dundee	55	2	25	5	4	5	4	5	2
Harrismith	69	14	23	9	6	16	7	0	0
Grant mean	191	5	21	6	3	9	4	2	2

Drinks were rarely wasted in all the towns. Milk was the mostly wasted drink in Richards Bay rural location and Dundee’s peri-urban location whist soft drink and juice were the most wasted drinks in Harrismith urban location (Table 7). The percentage of households wasting milk also followed the agro-ecological gradient although the differences in the percentages in Richards Bay and Dundee were minimal.

**Table 7:** Percentage of households wasting different drinks per town in the previous 48 hours.

Town	Milk	Soft drinks	Juice
Richards Bay	17	4	0
Dundee	16	4	2
Harrismith	7	9	9
Grand mean	13	6	4

### Quantities of food waste along the agro-ecological gradient

The overall mean quantity of food waste during the previous 48 hours was 121.0±132.4 g of prepared food (90% CI: 100.8 to 141.3 g), 268.6±610.1 g of unprepared food (90% CI: 175.5 to 361.7 g) and 77.0±192.5 ml of drinks (90% CI: 47.7 to 106.4 ml) per household (Table 8). Within sites, households in the urban locations generated a mean amount of prepared food waste of 99.2 g to 192.0 g in the last 48 hours, 89.5 g to 141.6 g in the peri-urban locations and 66.7 g to 141.1 g in the rural locations (90% CI) (Table 8). There were no significant differences in the amount of prepared food waste that was produced between towns (F_2, 113_ = 1.35, p = 0.26) nor between locations (F_2, 113_ = 0.90, p = 0.41).

**Table 8:** The mean amount of food wasted per household in the past 48 hours.

Town and location	N	Prepared food	SEM	90% LCL	90% UCL	Unprepared food	SEM	90% LCL	90% UCL	Drinks	SEM	90% LCL	90% UCL
Richards Bay	39	137.3±158.4	25.4	94.6	180.1	493.2 ^**a**^ ±965.1	154.5	232.6	753.7	63.8±177.7	28.5	15.9	111.8
*Urban*	11	168.8±211.0	63.6	53.5	284.1	106.1^**^±212.3	64.0	-10.0	222.1	18.6±39.0	11.8	-2.7	39.9
*Peri-urban*	16	106.0±107.0	26.7	59.1	152.8	326.5^**^±547.5	136.9	86.6	566.5	27.2±53.0	13.3	4.0	50.4
*Rural*	12	150.3±167.1	48.2	63.7	237.0	1070.2^*^±1488.1	429.6	298.7	1841.7	154.2±301.7	87.1	-2.2	310.6
Dundee	39	92.3±94.6	15.2	66.7	117.8	151.5 ^**b**^ ±311.6	49.9	67.4	235.7	65.4±173.6	27.8	18.5	112.3
*Urban*	11	83.2±101.8	30.7	27.6	138.8	150.0±310.1	93.5	-19.5	319.5	40.9±120.0	36.2	-24.7	106.5
*Peri-urban*	16	106.6±98.8	24.7	63.3	149.9	176.3±396.3	99.1	2.6	349.9	100.0±239.4	59.9	-4.9	204.9
*Rural*	12	81.6±87.8	25.3	36.1	127.1	120.0±178.3	51.5	27.6	212.4	41.7±99.6	28.8	-10.0	93.3
Harrismith	40	133.2±134.8	21.3	97.3	169.1	163.7 ^**b**^ ±198.2	31.3	110.9	216.5	101.3±223.7	35.4	41.6	160.9
*Urban*	13	178.8±154.0	42.7	102.7	255.0	266.9±238.7	66.2	148.9	384.9	238.5^*^±333.0	92.4	73.8	403.1
*Peri-urban*	16	134.4±120.4	30.1	81.6	187.1	136.0±159.6	39.9	66.0	205.9	37.5^**^±108.8	27.2	-10.2	85.2
*Rural*	11	77.6±120.7	36.4	11.6	143.5	82.2±155.5	46.9	-2.8	167.1	31.8^**^±90.2	27.2	-17.5	81.1
Grand mean	118	121.0±132.4	12.2	100.8	141.3	268.6±610.1	56.2	175.5	361.7	77.0±192.5	17.7	47.7	106.4
*Urban*	35	145.6±162.4	27.4	99.2	192.0	179.6±258.1	43.6	105.9	253.4	107.3±233.2	39.4	40.6	173.9
*Peri-urban*	48	115.6±107.6	15.5	89.5	141.6	212.9±401.0	57.9	115.8	310.0	62.2±158.5	22.9	23.8	100.6
*Rural*	35	103.9±130.3	22.0	66.7	141.1	433.9±975.5	164.9	155.1	712.7	67.1±192.4	32.5	12.2	122.1

* Values for prepared food, unprepared food and drinks are expressed as means ± SD (n = given in 2nd column).

LCL = Lower confidence limit; UCL = Upper confidence limit; SEM = Standard error of mean. Unlike superscripts indicate significant differences between towns (^a,b^) and between locations (*,**).

Data was analysed using two-way ANOVA, no significant differences were observed in the amount of prepared food waste between towns (F_2, 113_ = 1.35, p = 0.26) nor between locations (F_2, 113_ = 0.90, p = 0.41). For unprepared food: pairwise comparisons by post hoc Bonferroni indicated: 1. Amount of unprepared food waste (F_2, 113_ = 4.13, p = 0.019) in Richards Bay households (n = 39) was more (p<0.05) than that in Dundee households (n = 39) and in Harrismith households (n = 40), although it was similar in latter. 2. For Richard Bay town: Amount of unprepared food waste (F_2, 36_ = 3.74, p = 0.033) was more in rural households (n = 12) than the peri-urban (p < 0.05) (n = 16) and urban households (p < 0.05) (n = 11). For drinks waste in Harrismith town: pairwise comparisons indicated: 1. Amount of drinks waste (F_2, 37_ = 4.22, p = 0.022) in urban households (n = 13) was more (p<0.05) than in the peri-urban (p = 0.01, n = 16) and rural households (p = 0.02, n = 11).

Considering unprepared food, although the greatest percentage of households throwing away unprepared food was in Harrismith, with Richards Bay having the least, the mean amount of unprepared food waste was higher in Richards Bay with the mean amount of unprepared food waste being 493.2±965.1 g per household in the previous 48 hours (90% CI: 232.6 g to 753.7 g). There was a significant difference in the amount of unprepared food waste observed between the towns, (F_2, 113_ = 4.13, p = 0.019) and pair-wise comparisons (Bonferroni correction) for unprepared food waste have shown that Richards Bay households threw away unprepared food significantly more than Dundee households (p<0.05) and Harrismith households (p<0.05) (Table 8). No significant differences were observed in the mean amount of unprepared food waste between the locations (F_2, 113_ = 1.89, p = 0.156), being 179.6±258.1 g (90% CI; 105.9 g to 253.4 g) per household in the previous 48 hours in the urban locations, 212.9±401.0 g (90% CI: 115.8 g to 310.0 g) per household in the peri-urban location and 433.9±975.5 g (90% CI: 155.1 g to 712.7 g) per household in the rural locations (Table 8). However, significant differences were observed in Richards Bay between the amount of unprepared food wasted (F_2, 36_ = 3.74, p = 0.033), with rural households throwing away significantly more unprepared food than the urban households (p<0.05) and peri-urban households (p<0.05).

The mean amount of drinks wasted per household in the previous 48 hours was 77.0±192.5 ml (90% CI: 47.7 ml to 106.4 ml) and the amount of drinks wasted was relatively similar between towns (F_2, 113_ = 0.429, p = 0.653) (Table 8). Within sites, the mean amount of drink waste was 107.3±233.2 ml (90% CI: 40.6 ml to 173.9 ml) per household in the previous 48 hours in the urban locations, 62.2±158.5 ml (90% CI: 23.8 ml to 100.6 ml) in the peri-urban location and 67.1±192.4 ml (90% CI: 12.2 ml to 122.1 ml) in the rural locations (F_2, 113_ = 0.568, p = 0.569) (Table 8). However, significant differences were observed in Harrismith between the amount of drinks wasted (F_2,37_ = 4.22, p = 0.022) and post hoc tests showed that urban households were throwing away significantly more drinks than both the peri-urban households (p = 0.01) and rural households (p = 0.02). Furthermore, the estimated amount of food waste per household per year as well as the amount wasted per person per year in the study sites was extrapolated from the 48 hrs mean quantities as given in Table 9. No correlations were observed between different food waste types and household socio-economic characteristics, including household size, wealth, household food expenditure and HFIAS for most situations (S1 Table). However, there was a negative one between prepared food waste and household size, and a positive one between unprepared food waste and HFIAS in Richards Bay only (S1 Table).

**Table 9:** The overall estimate of the amount of food waste generated by each household and each person per year.

Food waste generation time frame	Prepared 90% LCL (g)	Prepared 90% UCL (g)	Unprepared 90% LCL (g)	Unprepared 90% UCL (g)	Drinks 90% LCL (ml*)	Drinks 90% UCL (ml*)	Grand range (g)
48hrs (g or ml)	101	141	176	362	48	106	325–609
*per household/year	18396	25787	32029	66010	8705	19418	59130–111215
**per person/year	2666	3737	4642	9567	1262	2814	8570–16118
per person/year in kg	2.7	3.7	4.6	9.6	1.3	2.8	8.6–16.1

Values denote the amount of food wasted per household and/ per person in a given time frame.

* Amount in 48hrs x 365/2.

** amount wasted per household/ mean household size (7 persons per household).

ml* were converted to kg using conversion factor of 1 litre:1 kg.

### Reasons for household food waste

The reasons given by the respondents regarding why they throw away the different foods in their households are enumerated in Table 10. The most cited reasons for the prepared food waste were that the food looked or smelt bad and that households had prepared too much and it was not possible for them to save leftovers. In Richards Bay, the highest reasons were because households had prepared too much and it was not possible for them to save leftovers and because the food had gone off/bad (Table 10). In Dundee, the two most common reasons for throwing away prepared food were that the food was off/bad and that they had prepared too much and it was not possible to save leftovers. In Harrismith, prepared food was wasted mostly because the food was off/bad or the respondent had served too much and could not finish all the food (Table 10).

**Table 10:** Most cited reasons by households for throwing away food at each study site.

Food wasted	Reason for throwing away food	Richards Bay	Dundee	Harrismith	All
Prepared food	Prepared too much and not possible to save leftovers	34	26	6	22
	Prepared too much and do not want to save leftovers	11	5	14	10
	Served too much and could not finish all	11	7	16	12
	Saved leftovers but were not used in time	8	10	4	7
	Food was burnt/ruined during cooking/preparation	6	12	8	8
	The food did not taste nice	2	5	14	7
	Food visibly bad or smelt bad	19	29	22	23
Unprepared food	Passed best before date	50	36	49	45
	Bought too much	0	14	3	7
	Food has gone bad (rotten, sour or moldy)	36	41	36	38
Drinks	Passed best before date	33	83	60	59
	Accident	53	0	27	27

Note: All values in the table are presented as %.

For unprepared food, households in all towns threw away food mostly because the food had passed its best before date or the food was bad i.e. rotten, sour or moldy (Table 10). A smaller percentage of households, especially in Dundee had bought too much and has ended up throwing away the unprepared food. In general, the greatest percentage of households wasted drinks when they had passed the best before date or by accident. Drinks were mostly wasted in Dundee and Harrismith when these had passed the best before date whilst in Richards Bay it was mainly due to accidents although a greater percentage of households had thrown away the drinks that had passed the best before date (Table 10). Also, a greater percentage of the households in Dundee had wasted drinks by accident (Table 10).

## Discussion

Results from this study suggest that households in all three towns were showing signs of minimising the amount of food they threw away as more than 80% of the households in all towns consume their meals at home with all household members, and rarely left any food uneaten. If they happened not to finish the food, they usually kept it and consumed it in the next day or two. This corroborates other studies reporting that minimal food waste occurs when household members eat together at home (Lebersorger (cited in [38])) rather than eating out. Only 35% of the sampled households had thrown away any food in the previous 48 hours and the greatest percentage had thrown away prepared food, especially pap, meat and vegetables (mostly cabbage) and, to a lesser extent, drinks. The type of food that was wasted depended on the food types that were being consumed by households as more than 50% of households across all towns and locations consumed starchy cereals (mostly maize meal), vegetables (mostly cabbage and onion) and meat [39]. This is slightly different from the types of food that were reported to have been wasted in the developed world where Thönissen [40] found a high proportion of dairy products being wasted in the Netherlands and Pekcan et al. [41] reported the highest proportion of food waste in Turkey consisted of fresh fruits and vegetables. However, the wasting of meat is consistent with other studies, especially in the developed world as meat has also been reported as one of the products contributing to food waste in the UK [19], the Netherlands [40], Austria (Lechner and Schneider (cited in [11])), USA [42] and in Turkey [41]. In South Africa, meat and fish are widely consumed [43], therefore, it was easier for households in the present study to generate food waste from meat as it is readily available in the households. Globally, fruits and vegetables, starchy cereals, fish, meat and dairy contribute more than 20% of food waste per annum [6–7].

The percentages of households who were throwing away food and drinks did not differ along the agro-ecological gradient. However, location along the rural-urban continuum did correlate with the percentage of households throwing away drinks as was shown by the significantly higher prevalence of wasting drinks in urban locations relative to both peri-urban and rural locations. Most drinks thrown away in the urban locations were soft drinks and juice, whilst in the peri-urban and rural locations it was more commonly milk. This could be attributed to households in urban areas having good access to cheap and affordable goods which can encourage bulk buying, some of which may end up expiring before being used. Milk was mostly wasted in rural and peri-urban areas because it is a perishable product and requires proper storage and cooling, which can be lacking in poor rural households.

In general, households generated greater quantities of unprepared food waste (268.6±610.1 g) than prepared food waste (121.0±132.4 g); t(234) p = 0.011 as was hypothesised in the study (hypothesis four). No significant differences were found in the amounts of prepared food waste and drinks waste between and within the towns. Although there was no significance difference across towns for prepared food waste, the amount of unprepared food waste followed the agro-ecological gradient as per the study hypothesis one with households in Richards Bay throwing away greater quantities (493.2±965.1 g per household per 48 hours) of unprepared food than the other two towns. When extrapolating the findings from the 48 hour-period to over a year; households wasted approximately 32.0–66.0 kg (90% CI) of unprepared food per year, equating to approximately 4.6–9.6 kg per person per year of unprepared food waste (when using mean sample household size). Households in the study sites also waste approximately 18.4–25.8 kg (90% CI) of prepared food per household per year and 2.7–3.7 kg per person per year and 8.7–19.4 litres (90% CI) of drinks per household per year with each household member wasting approximately 1.3–2.8 litres of drinks annually. The estimated per capita food waste by consumers in this study (8.6–16.1 kg per person per year), including unprepared, prepared and drinks waste, overlaps with that which is estimated in the developing countries (6–11 kg per person per year in sub-Saharan Africa and South/Southeast Asia) and lower than that in the developed countries (95–115 kg per person per year in Europe and North America) as reported by Gustavsson et al. [6]. Although there is an overlap between the estimated per person per year food waste quantities for this study and that for developing countries, the average per capita food waste by consumers for the present study which is 12.35 kg ((8.6+16.1)/2) is higher than that for developing countries which is 8.5 kg ((6+11)/2), reflecting South Africa’s middle-income status. However, further studies need to be done in these areas to measure the quantities of food waste before concluding on the actual figures of the quantities of food that is being discarded per annum.

The amount of unprepared food waste differed between towns, being higher in Richards Bay than the other two. This could be because of high levels of food access in Richards Bay [39] which could be attributed to wetter and warmer climatic conditions and a longer growing season which favours agriculture. The same weather conditions can also affect the processing and storage of the produce which could promote rotting as this was one of the reasons why about 36% of the households in Richards Bay threw away unprepared food. Lack of infrastructure and associated technical and managerial skills in food production and post-harvest processing have been reported as the main driver promoting food waste in developing countries, although this might apply on a large scale [11,44]. Also, food waste in developing countries has been linked to poor financial status, storage and cooling facilities [6] and most food was wasted in the rural areas in Richards Bay where most households fall into low income status [39], have limited market and knowledge on how to preserve their farm produce [39] and could have poor storage and cooling facilities.

A significant negative correlation between the amount of prepared food waste and household size was observed, i.e. smaller households were wasting more prepared food than larger households, as was hypothesised in the study (hypothesis six). This could be because small households prepare large portions of food which they failed to eat as they generously spend their resources which may appear as more than enough. In larger households, resources may appear as insufficient, therefore, members may be sparing their resources through exercising portion measurements and can only prepare what would be enough for the meal. These results are consistent with studies from developed countries which have shown that larger households waste less food per person than smaller households [11,18–19]. Also, a significant positive correlation in the amount of unprepared food waste and HFIAS was observed in Richards Bay meaning that households with poor food access were discarding greater amounts of unprepared food than those who had good access to food, which was opposite to what had been hypothesised in the study (hypothesis five). Households with poor food access could have been bulk buying when food was being sold at lower prices when they had the resources to acquire food and the food passed the expiry date before being used and for perishable food, the quality could have been poor and the food spoilt before being used. In other studies in the developed countries, the availability of cheap food (which may increase household access to food) has been noted to encourage overbuying and hoarding behaviours that result in food waste [16]. That is, impulse buying as a result of retail promotions, poor storage practices which results in food becoming moldy or ‘off’ and poor food management in homes where food is not used before going past ‘use by’ or ‘best before’ date has also been reported in the UK [45]. This also applies to prepared food where a large percentage of households in all towns discarded the food because it had gone bad and more drinks were discarded because they had passed the best before date. Households also prepared large portions of food which they ended up not eating and although they could have served leftovers, they could not use them on time. This is consistent with Exodus [45] who reported poor portion control as households in UK prepared meal portions that were too large resulting in an inability to finish all the food.

There have been reports that low-income households throw away less food than high-income households [21]. However, the present study showed no significant associations between the quantities of food waste (prepared, unprepared and drinks) with household socio-economic status indices (food expenditure and wealth index) as was also reported Parfitt et al. [11]. This could be because households which were sampled had a narrow difference in the wealth. However, further research need to be done to fully support this finding. In general, urban households in Harrismith wasted more food than the rural and peri-urban households which could be because they had more access to food. Also, greater quantities of drinks were wasted in urban locations than in the peri-urban and rural locations, which can also point to the issue of affordability, i.e. urban households have a higher socio-economic status and can afford to buy drinks in larger quantities than peri-urban or rural households. However, this was not consistent in the other towns as more food was wasted in the rural locations in Richards Bay and in the peri-urban locations in Dundee.

## Conclusion

More households in this study were discarding prepared food than unprepared food and drinks but the quantities of unprepared food discarded were significantly higher than prepared food. Quantities of unprepared food waste followed the agro-ecological gradient with residents in Richards Bay discarding greater quantities than the other towns. Households in the study sites waste approximately 32–66 kg of unprepared food per year with each member wasting approximately 5–10 kg per year. The average estimated per capita food waste by consumers in this study, including all food waste types, is higher than that which is estimated in the developing countries (average 8.5 kg/person/year in sub-Saharan Africa and South/Southeast Asia) and lower than that in the developed countries (average 105 kg/person/year in Europe and North America). Household food waste in the study sites was mainly a result of household behavior concerning food preparation (as the majority of the households threw away food because they could have prepared too much and not possible to save left overs) and storage (as food became visibly bad and smelly bad) as was noted in the developed countries [6,46]. Since many households in this study were preparing too much food which they ended up discarding, integrated approaches are required to address this issue affecting South African societies, which include promoting sound food management to decrease household food waste. Nonetheless, further studies need to be conducted to fully understand the reasons why households prepare too much food and yet they do not like to consume leftovers. In this case, one can conclude if there is need for increased awareness on measuring ingredients when preparing food so that households cook portion sizes which can all be eaten and can also make use of leftovers to make new meals. Also, education campaigns focusing on raising awareness on consumer food purchasing skills, meal planning, using leftovers into new meals, interpreting sell-by, use-by and best before dates as well as food management and storage skills so that food can have a longer life even on the shelves [46–47], should not be ignored. This may also apply to South Africa as some of the food in the study sites was discarded because it had passed best before date, had gone bad (rotten, sour or moldy), and some households thought it was not possible to save left overs. One of the biggest gaps in South Africa lies in the awareness and knowledge of food waste in the food system [24]. In areas like Richards Bay where households practice agriculture, campaigns should focus on supporting households on how to process their produce, especially drying vegetables after harvesting, which they can use in the future. This may decrease reports on the cases where food may go bad/become rotten hence reducing the amount of food being thrown away. All the above-mentioned recommendations need to be tested so as to understand if it can help households to minimise the quantities of food waste they generate. In the South Africa, costs associated with disposal of household food waste to landfill are estimated at R505 million per annum [25]. Considering the rising food prices and global food shortages, reducing food waste significantly increases water and food security in many parts of the world as well as reducing greenhouse gas emissions, conserving energy, protecting soil from degradation and decreasing pressure for land conversion into agriculture [3,48].

## Supporting information

S1 Table Spearman correlations between HFIAS, household size, food expenditure and wealth status of households with the amount of food wasted by households in the previous 48 hours. The correlations between different food waste types (prepared and unprepared food waste and drinks waste) and household socio-economic characteristics, including household size, wealth, household food expenditure and HFIAS. The Significant correlations at p<0.05 are shown in bold.
(DOCX)

S1 File Food and Nutrition questionnaire. The questionnaire that was used to capture information on household food waste in South Africa. The specific questions on food waste are on section D of the questionnaire.
(PDF)

S2 File Information to participants. All the information about the project that was presented to prior to data collection is in the information to participants file. Participants were first informed about the project and were asked for their willingness to participate. Once they had agreed to participate, they would sign consent forms.
(PDF)
