# Supplementary material for: Local setting influences the quantity of household food waste in mid-sized South African towns
Source: PLoS One. 2017 Dec 12;12(12):e0189407. doi: 10.1371/journal.pone.0189407 (PMC5726726; doi:10.1371/journal.pone.0189407)
Supplement: S1 Table — The correlations between different food waste types (prepared and unprepared food waste and drinks waste) and household socio-economic characteristics, including household size, wealth, household food expenditure and HFIAS. The Significant correlations at p<0.05 are shown in bold. (DOCX) [file pone.0189407.s001.docx]

Table 1. Spearman correlations between HFIAS, household size, food expenditure and wealth status of households with the amount of food wasted by households in the previous 48 hours.

| Food waste type | Town | | HFIAS | Household size | Food expenditure | Wealth |
| --- | --- | --- | --- | --- | --- | --- |
| Prepared | | Richards Bay | 0.14 | -0.09 | 0.02 | -0.16 |
|  |  | Dundee | 0.07 | -0.3 | 0.02 | -0.2 |
|  |  | Harrismith | -0.2 | -0.2 | 0.05 | 0.12 |
|  |  | All | 0.04 | **-0.23** | 0.10 | -0.06 |
| Unprepared | | Richards Bay | **0.51** | -0.16 | -0.07 | 0.16 |
|  |  | Dundee | 0.2 | -0.01 | -0.12 | -0.01 |
|  |  | Harrismith | 0.03 | 0.19 | 0.21 | 0.08 |
|  |  | All | 0.18 | 0.09 | -0.12 | -0.02 |
| Drinks | | Richards Bay | -0.08 | 0.17 | -0.09 | 0.26 |
|  |  | Dundee | 0.49 | 0.21 | -0.25 | 0.30 |
|  |  | Harrismith | -0.4 | -0.17 | 0.03 | -0.2 |
|  |  | All | 0.10 | 0.11 | -0.01 | 0.18 |

Bold correlations are significant at p<0.05.
