# Supplementary material for: Local setting influences the quantity of household food waste in mid-sized South African towns
Source: PLoS One. 2017 Dec 12;12(12):e0189407. doi: 10.1371/journal.pone.0189407 (PMC5726726; doi:10.1371/journal.pone.0189407)
Supplement: S1 File — The questionnaire that was used to capture information on household food waste in South Africa. The specific questions on food waste are on section D of the questionnaire. (PDF) [file pone.0189407.s002.pdf]

Food and Nutrition Questionnaire

IMPORTANT: Interview woman of reproductive age (15 – 49 years) preferably!

Date________________ **Researcher/Enumerator**______________________________

Sampling ID (e.g. SA_RIC_001 or SA_DUN_001 or SA_HAR_001) ___________________

Name of suburb or section____

Alternative household (in case originally sampled household is not available)
◯ 1st house to the left ◯ 2nd house to the left ◯ 3rd house to the left

A Household Background Information

A1 Interviewee

Age of interviewee (in years) __________**Sex of interviewee** ◯ male ◯ female

Highest education grade attained of interviewee________________________________

Main economic occupation
◯ none ◯ housewife ◯ owner farmer ◯ farm wage labourer
◯ non-farm wage labourer* ◯ salaried job* ◯ own business* ◯ other*

***please specify:** _____________________________

Household head if different from the interviewee

Age of household head (in years)___________**Sex of household head** ◯ male ◯ female

Highest education grade attained of household head____________________________

Main economic occupation
◯ none ◯ owner farmer ◯ farm wage labourer ◯ non-farm wage labourer*
◯ salaried job* ◯ own business* ◯ Pensioner/ Retired ◯ other*

***please specify:** _____________________________

A2 How many people live together with you in this household? (By "household" we mean those of you that sleep under the same roof and take meals together at least four days a week)

**Sex**	**adults ≥18 years**	**children (6-17 years)**	**Small children (≤ 5 years)**
**Female**			
**Male**			

A3 How many adults in the HH work full-time (self-employed or employed) for cash remuneration? Males_____ Females______

A4 How many adults in the HH work part-time or casually (for themselves or employed) for cash remuneration? Males_____ Females______

A5 How many of the following does your household own?

Car/Truck		Tractor		TV		Fridge		Cattle/Goats		House	
Motorbike		Bicycle		Radio		Cell phone		Chicken			

A6 Does the house you live in have electricity? ◯ yes ◯ no

A7 How many rooms does the house have? _______

A8 How long have you lived in this house? Years________ OR Months_______

A9 How long have you lived in this neighbourhood or village? Years___ OR Months___

A10 If less than 10 years, from where did you move?

◯ Same neighbourhood/village/quarter ◯different neighbourhood/quarter in same town/city
◯ Another place in the same province ◯Another place in a different province

B Aspects of Household Food Acquisition

B1 How often do you normally obtain food from these sources?

	At least once a week	At least once a month	At least once in every six months	Less than once a year	Never
Own production in the town					
Own production in the outskirts of town					
Own production in the rural areas					
Purchase from general food markets					
Purchase from small retail, kiosk or fast food outlets					
Purchase from supermarket(s)					
Purchase from door-to-door salesman					
Collected from open spaces in town					
Collected from open spaces on outskirts of town					
Collected from open spaces in rural areas					
Donated by family/relatives					
Donated by friends					
Donated by government or NGOs					

B2 If your household is doing own food production, how much land do you cultivate?

Number___ Unit ◯ ha ◯ acre ◯ m²

B3 Approximately how much does your household normally spend on food purchases per week?

Amount_____ Currency ◯ ZAR

B4 Estimate the proportion of your household food needs that are obtained from own-production

**Own production**	**None**	**Less than half**	**More than half**	**All**
Staple foods				
Vegetables				
Fruits				
Milk (dairy)				
Meat				
Fish				
Eggs				
Others (specify)				

B5 Estimate the proportion of your household food needs that are obtained from wild collected foods

**Wild collected foods**	**None**	**Less than half**	**More than half**	**All**
Vegetables				
Fruits				
Meat				
Fish				
Edible insects				
Others (specify)				

C Assessment of Household Food Security by using the Household Food Insecurity Access Score (HFIAS) Tool

C1 In the past four weeks, did you worry that your HH would not have enough food? If yes, how often did this happen?

◯ No ◯ Yes, rarely (once or twice in the past four weeks)

◯ Yes, sometimes (three to ten times in the past four weeks)

◯ Yes, often (more than ten times in the past four weeks)

C2 In the past four weeks, were you or any HH member not able to eat the kinds of foods you preferred because of lack of resources ("lack of resources" means not having the means to get food, either for growing it, purchasing it or trading for it)? If yes, how often did this happen?

◯ No ◯ Yes, rarely (once or twice in the past four weeks)

◯ Yes, sometimes (three to ten times in the past four weeks)

◯ Yes, often (more than ten times in the past four weeks)

C3 In the past four weeks, did you or any household member have to eat a limited variety of foods due to a lack of resources? If yes, how often did this happen?

◯ No ◯ Yes, rarely (once or twice in the past four weeks)

◯ Yes, sometimes (three to ten times in the past four weeks)

◯ Yes, often (more than ten times in the past four weeks)

C4 In the past four weeks, did you or any household member have to eat some foods that you really did not want to eat because a lack of resources to obtain other types of food? If yes, how often did this happen?

◯ No ◯ Yes, rarely (once or twice in the past four weeks)

◯ Yes, sometimes (three to ten times in the past four weeks)

◯ Yes, often (more than ten times in the past four weeks)

C5 In the past four weeks, did you or any household member have to eat a smaller meal than you felt you needed because there was not enough food? If yes, how often did this happen?

◯ No ◯ yes, rarely (once or twice in the past four weeks)

◯ Yes, sometimes (three to ten times in the past four weeks)

◯ Yes, often (more than ten times in the past four weeks)

C6 In the past four weeks, did you or any household member have to eat fewer meals in a day because there was not enough food? If yes, how often did this happen?

◯ No ◯ yes, rarely (once or twice in the past four weeks)

◯ Yes, sometimes (three to ten times in the past four weeks)

◯ Yes, often (more than ten times in the past four weeks)

C7 In the past four weeks, was there ever no food to eat of any kind in your household because of lack of resources to get food? If yes, how often did this happen?

◯ No ◯ yes, rarely (once or twice in the past four weeks)

◯ Yes, sometimes (three to ten times in the past four weeks)

◯ Yes, often (more than ten times in the past four weeks)

C8 In the past four weeks, did you or any household member go to sleep at night hungry because there was not enough food? If yes, how often did this happen?

◯ No ◯ yes, rarely (once or twice in the past four weeks)

◯ Yes, sometimes (three to ten times in the past four weeks)

◯ Yes, often (more than ten times in the past four weeks)

C9 In the past four weeks, did you or any household member go a whole day and night without eating anything because there was not enough food? If yes, how often did this happen?

◯ No ◯ yes, rarely (once or twice in the past four weeks)

◯ Yes, sometimes (three to ten times in the past four weeks)

◯ Yes, often (more than ten times in the past four weeks)

D Individual Dietary Diversity (only for women of between 15 - 49 years)

D1 What is your relationship to the head of household?

wife		sister		niece		mother	
daughter		grand daughter		aunt		Other*	

*specify_____________________

D2 Did you eat anything (meal or snack) outside of the home during the last 48 hours (i.e. yesterday or the day before yesterday)?
◯ yes ◯ no

D3 Did your food differ during the last 48 hours (yesterday and the day before yesterday) from your usual diet due to a special reason? (e.g. birthday, wedding ceremony, etc.)?
◯ yes ◯ no

D4 Were you sick yesterday?
◯ yes ◯ no

D5 Age of respondent in years_________

D6 Please describe the foods (meals and snacks) and drinks that you consumed during the past two days day and night at home and the food that was not eaten.

D6 (a) Food and drinks consumed yesterday

Meal	Ingredients	*Source of ingredients	*Source of energy
**Before breakfast**			
**Drinks**			
**Breakfast**			
**Drinks**			
Was all the food prepared for breakfast eaten?		Yes	No
If not, approximately how much was left? (** cup-full **)			
If not, why was it not all eaten? (** codes **)			
If not, what was done with the remainder? (** codes **)			
**Snacks before lunch**			
**Drinks**			
**Lunch**			
**Drinks**			

Was all the food prepared for lunch eaten?		Yes	No
If not, approximately how much was left? (* cup-full *)			
If not, why was it not all eaten? (* codes *)			
If not, what was done with the remainder? (* codes *)			
**Snacks before dinner**			
**Drinks**			
**Dinner**			
**Drinks**			
Was all the food prepared for dinner eaten?		Yes	No
If not, approximately how much was left? (* cup-full *)			
If not, why was it not all eaten? (* codes *)			
If not, what was done with the remainder? (* codes *)			
**Snacks after dinner**			
**Drinks**			

D6 (b) Food and drinks consumed the day before yesterday

Meal	Ingredients	*Source of ingredients	*Source of energy
**Before breakfast**			

**Drinks**			
**Breakfast**			
**Drinks**			
Was all the food prepared for breakfast eaten?		Yes	No
If not, approximately how much was left? *(* cup-full *)*			
If not, why was it not all eaten? *(* codes *)*			
If not, what was done with the remainder? *(* codes *)*			
**Snacks before lunch**			
**Drinks**			
**Lunch**			
**Drinks**			
Was all the food prepared for lunch eaten?		Yes	No
If not, approximately how much was left? *(* cup-full *)*			
If not, why was it not all eaten? *(* codes *)*			
If not, what was done with the remainder? *(* codes *)*			

**Snacks before dinner**			
**Drinks**			
**Dinner**			
**Drinks**			
Was all the food prepared for dinner eaten?		Yes	No
If not, approximately how much was left? (** cup-full **)			
If not, why was it not all eaten? *(* codes *)*			
If not, what was done with the remainder? *(* codes *)*			
**Snacks after dinner**			
**Drinks**			

***D7 Dietary Diversity Score (DDS) ________   * Quantity wasted _________**

E Care Giving Practices including Breast Feeding

Interview a mother/caregiver of a child of between 6 months to 2 years old. If there is no child of that age group, skip the section. Throughout the following section make sure that you only talk about one particular child.

E1 Name of the child (only if child is between 6 months and 2 years)

____

E2 What is the age of your child? (in full months only**)**______________________

E3 During your pregnancy with above mentioned child, how many times did you visit a health care facility for a prenatal visit? ________________________

E4 Did you ever breastfeed the above mentioned child?

◯ yes ◯ no

E5 How many hours after birth did you start breastfeeding the above mentioned child? ________

E6 Did you feed colostrum (the breast milk produced in the first 3 days after birth) to the above mentioned child?

◯ yes ◯ no

E7 During the first 3 days after birth, was the above mentioned child given anything other than breast milk?

◯ yes ◯ no

E8 If yes, what was it? ____

End of survey: Thank you very much for your participation!

Would you be willing to participate in further research related to food security and nutrition?

◯ yes ◯ no

Do you have any questions for us?
