# Supplementary material for: Local setting influences the quantity of household food waste in mid-sized South African towns
Source: PLoS One. 2017 Dec 12;12(12):e0189407. doi: 10.1371/journal.pone.0189407 (PMC5726726; doi:10.1371/journal.pone.0189407)
Supplement: S2 File — All the information about the project that was presented to prior to data collection is in the information to participants file. Participants were first informed about the project and were asked for their willingness to participate. Once they had agreed to participate, they would sign consent forms. (PDF) [file pone.0189407.s003.pdf]

RHODES UNIVERSITY
Where leaders learn

Grahamstown ● 6140 ● South Africa

Information to participants.

My name is _____________________. I am a student at Rhodes University (Grahamstown) and I am doing a research on the issue of food security and waste which is becoming a problem in this country. You are invited to be part of this research and I am going to give you information about this research. Please ask me to stop as we go through the information and I will take time to explain. If you have questions later, you can ask me and I will answer them.

This research is about food security and food waste. I would like to know where you get the food you eat and the types of the food you eat. I also need to know the names of the food you eat each day and if there are any signs of missing some meals or eating what you do not like. The challenges you have in acquiring the food you eat. This information will help us to identify households and household members who maybe food insecure in your communities. I would also need to know if there are times when you feel you have plenty of food and if there are times when you through away food in your households. Be it food that you have cook, the raw food and drinks including milk and juice. If there is food which you do not feel like eating and want to throw it away, may you please measure and record it for the next 48hrs and list which food you have discarded and why you did so. You can use standard household measures such as cups, tablespoons or teaspoons to measure the quantity of food you throw away. I can demonstrate to you how you can do this and if you have any questions while I am doing it, you can stop me and I can explain again until you know what you should do. I will come back in the next two days and do a questionnaire interview with you which will include questions on both food security and food waste. I will be asking you to answer some questions on the questionnaire regarding what I have mentioned above.

If you have a child who is under the age of five years, I will also ask for your permission to take his/her standing height and mid-upper arm circumference measurement. I will visit you to do interviews three times in the first season and once in the second season to compare if there are any changes in your diet. The first interview will be approximately 45-60 minutes and the last three will be 20-30 minutes. If you are selected to take part in a focus group discussion, it will only last for an hour.

During the interviews and group discussions, you do not need to give me your name. Any information about you and your household will have a number on it which can only be linked to you by me. I will lock that information up and it will not be shared with or given to anyone except my supervisor. I will not be sharing information about you to anyone and the

information that I collect from this research project will be kept private. Your identity will not be revealed in any publication resulting from this study.

This research is an academic research and findings from this research will help in identifying households that are vulnerable to food insecurity and information will be communicated to responsible authorities for intervention. The study will also give recommendations to policy makers to consider small and medium sized populations in their countrywide food security policies. Participation in this research might inconvenience or distress participants in the case of living in poverty or food insecure. However, responsible authorities will be informed to give counselling and help the household, for example by providing food parcels only if participants allow us to and if there are offices practicing that in the town.

You have the right to decide not to participate in this research, to withdraw from participating in the project at any time, even after you have given consent and after the project commences or even during the interview. You are free to stop the interview if you feel like you no longer want to participate and the information given will be kept if you agree or destroyed before the researcher leaves your premises. The data from this research project will be stored and may not be used for a different purpose in future without obtaining a new consent from you. Results from this project will be communicated to you at the end of this project by post as information sheets simplifying a summary of the findings in your preferred language. To those with access to internet, results will also be sent for publication in academic journals.
